# Clinical value of heparin-binding protein in adult bacterial intracranial infection

**DOI:** 10.3389/fcimb.2024.1439143

**Published:** 2024-11-14

**Authors:** Linsai Guan, Feiyao Wang, Jingni Chen, Yanxin Xu, Weixing Zhang, Jianping Zhu

**Affiliations:** ^1^ Department of Nursing, Shanghai Taikang Shenyuan Rehabilitation Hospital, Shanghai, China; ^2^ Department of Critical Care Medicine, Shanghai General Hospital, Shanghai Jiao Tong University School of Medicine, Shanghai, China; ^3^ Department of Geriatrics, Shanghai General Hospital, Shanghai Jiao Tong University School of Medicine, Shanghai, China

**Keywords:** heparin binding protein, cerebrospinal fluid, intracranial infection, gram - negative bacteria, gram - positive bacteria

## Abstract

**Background:**

The accurate and sensitive diagnosis of intracranial infection continues to pose a critical challenge. This study aimed to probe into the clinical value of heparin binding protein (HBP) in bacterial intracranial infection.

**Methods:**

Patients suspected of having bacterial intracranial infection and admitted to Shanghai General Hospital from November 2021 to November 2023 were selected as study subjects and divided into an infected group and a non-infected group. The receiver operating characteristic (ROC) curve was constructed to compare the diagnostic accuracy of HBP, procalcitonin (PCT), and C-reactive protein (CRP), as well as their value in differentiating Gram-positive bacteria and Gram-negative bacterial infections.

**Results:**

According to the results of bacterial identification, the infected groups were divided into a Gram-negative bacteria group (n = 142) and a Gram-positive bacteria group (n = 128), while the non-infected group comprised 120 patients after neurosurgery involving dura opening. Statistically significant differences were observed in the levels of HBP, PCT, and CRP between the infected group and the non-infected group (all p< 0.05). Receiver operating characteristic (ROC) curve analysis showed that the area under the curve (AUC) of HBP was 0.935, and the AUCs of PCT and CRP were 0.931 and 0.863, respectively. In the comparison of HBP, PCT, and CRP levels in the Gram-negative bacteria and Gram-positive bacteria groups, the AUCs were 0.816, 0.602, and 0.591, respectively. When the cutoff value of HBP was 72.34 ng/mL, its specificity reached 96.1% and its sensitivity was 57.8%. When PCT and CRP levels were less than 1.67 ng/mL and 23.12 ng/mL, respectively, both the sensitivity (52.3%, 53.1%) and specificity (66.9%, 59.9%) were relatively low.

**Conclusion:**

HBP, PCT, and CRP can be employed as diagnostic indicators for bacterial intracranial infection. HBP (>72.34 ng/mL) can act as an important index for the diagnosis of Gram-negative bacteria in patients with intracranial infection.

## 1 Introduction

Intracranial infection, which includes meningitis, encephalitis, and brain abscess, is a common complication after neurosurgery and can also occur spontaneously (Glynn et al., 2023; Chen et al., 2024). For neurosurgical patients, although preoperative preventive use of antibiotics and standardized operations can reduce the incidence of intracranial infection, due to the invasive nature of central nervous system (CNS) procedures and increased use of implants, the incidence of intracranial infection after neurosurgery remains between 1.4% and 9.5% (Kim et al., 2018). CNS infection leads to prolonged hospital stays, increased costs, poor prognoses, and even death. Therefore, timely detection and treatment are extremely important (Giovane and Lavender, 2018; Wall et al., 2021; Hasbun, 2022; GBD 2021 Nervous System Disorders Collaborators, 2024). Acute intracranial infections present nonspecific symptoms and lack typical features during the infection process. Thus, a high degree of suspicion is needed for diagnosis (Michelson et al., 2024; Mohanty et al., 2024). The most common antibiotic-resistant bacteria associated with intracranial infection are Neisseria meningitidis, Staphylococcus aureus, Acinetobacter baumannii, Streptococcus pneumoniae, and coagulase-negative Staphylococcus (CoNS) (Nau et al., 2020; Chen et al., 2024).

Intracranial infection is one of the indications for emergency lumbar puncture tests and microbial culture (Völk et al., 2023). Prior to treating any infection, a biomarker can be utilized to distinguish between viral and bacterial infections. Cerebrospinal fluid (CSF) levels of HBP can aid in determining whether meningitis is caused by bacterial or viral infection (Obreja et al., 2022). Currently, CSF culture remains the gold standard for identifying the pathogens causing intracranial infection. However, waiting for culture results takes a long time, thus delaying the targeted treatment time (Hasbun et al., 2017; Poplin et al., 2020). Heparin-binding protein (HBP), also known as cationic antimicrobial protein of 37 kDa (CAP37) or azurocidin, is a multifunctional inactive serine protease homolog. The present research shows that HBP is released from neutrophils upon stimulation with secretagogues that do not trigger the secretion of azurophilic granule contents (Tapper et al., 2002). Owing to its significant bactericidal activity, chemotaxis, and inflammatory regulation, it increases significantly in the early stage of infectious diseases, which is beneficial for the diagnosis of infectious diseases (Tian et al., 2021; Wu et al., 2021). Nevertheless, few studies have explored the expression of HBP in patients with intracranial infection (Linder et al., 2011; Kong et al., 2022). CAP37 was initially recognized for its potent antibiotic activity against gram-negative bacteria and was regarded as a component of the oxygen-independent killing mechanism of neutrophils (Pereira, 1995). Therefore, elevated HBP levels in cerebrospinal fluid may serve as a predictor of intracranial gram-negative infection. Hence, we hypothesize that increased HBP levels in cerebrospinal fluid are closely related to intracranial gram-negative bacterial infection. This study aims to test this hypothesis.

Serum procalcitonin (PCT) is a highly accurate diagnostic test that physicians can use for rapid differentiation between bacterial and viral causes of meningitis in adults (Vikse et al., 2015; Ivaska et al., 2024). C-reactive protein (CRP) is markedly elevated during inflammatory conditions, establishing it as a prototypical acute phase protein that plays a role in innate immune responses (Norman-Bruce et al., 2024; Zhou et al., 2024). By comparing the levels of HBP, PCT, and CRP, this study evaluates the diagnostic value of HBP in Gram-negative bacteria intracranial infection.

## 2 Methods

### 2.1 Research object

**Figure 1:**
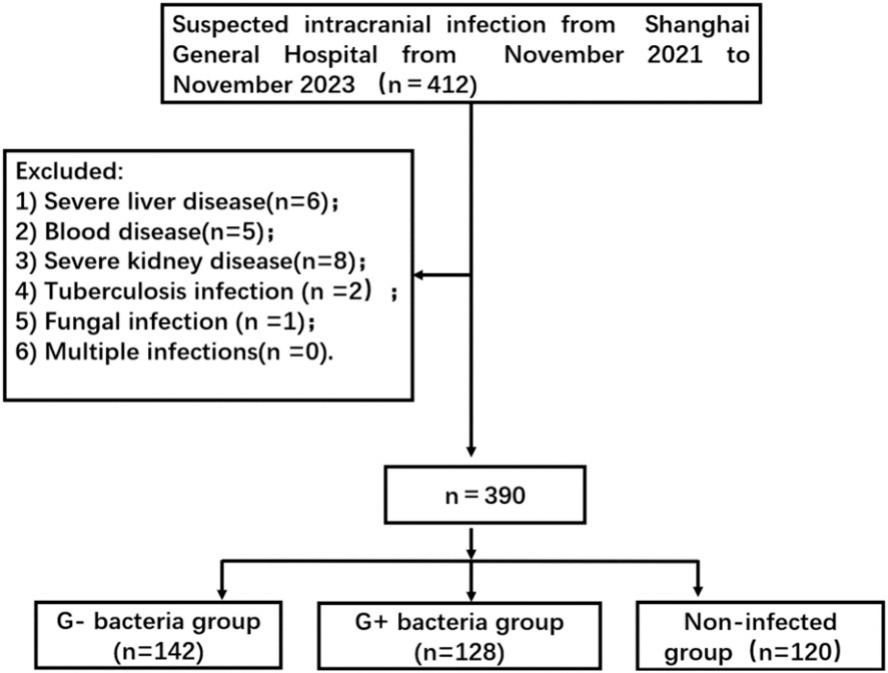
Flowchart of patient selection. G- bacteria group: Gram-negative bacteria group; G+ bacteria group: Gram-positive bacteria group.

For this prospective observational study, 390 patients with suspected intracranial infection who were admitted to Shanghai General Hospital from November 2021 to November 2023 were selected. Eventually, 270 of these patients were diagnosed with intracranial infection. The types of pathogenic bacteria were divided into two groups: a Gram-negative bacteria group (n = 142) and a Gram-positive bacteria group (n = 128) (**Figure 1**). The non-infected group consisted of 120 patients who had undergone neurosurgery involving dura opening. This study was approved by the ethics committee of Shanghai General Hospital 【2022KY052】. Each procedure was in accordance with the Helsinki Declaration.

### 2.2 Inclusion and exclusion criteria

Patients meeting all of the following inclusion criteria were included: (1) aged 18 years or older; (2) voluntarily participated in the study and provided informed consent; (3) underwent neurosurgery involving dura opening; and (4) as determined by the clinician, the patient might have an intracranial infection.

The exclusion criteria were as follows: (1) patients diagnosed with intracranial infection at other centers prior to admission; (2) patients with tuberculosis infection, infectious diseases such as viruses and fungi, and immune system diseases; (3) patients with severe dysfunction of the kidney, liver, or other organs or those with malignant tumors; and (4) patients with multiple intracranial infections. Patients who received empiric antibiotic therapy before sampling were not excluded.

### 2.3 Diagnostic criteria

Clinical features alone cannot be utilized to determine the presence of intracranial infection. Intracranial infection is diagnosed by conducting a spinal tap to obtain a sample of the fluid surrounding the brain and spinal cord. Newer technologies, such as genetic sequencing and multiplex polymerase chain reaction (PCR), may help provide quicker and more accurate diagnoses. In this study, next-generation sequencing (NGS) of cerebrospinal fluid was carried out with the consent of the patient or their family.

Intracranial infection was confirmed when a patient met criterion 1 or 2 of the definition: 1. A positive culture of CSF or confirmed diagnosis by NGS results; 2. At least one of these clinical symptoms that cannot be otherwise explained by other causes (body temperature > 38°C, headache, meningeal signs, or focal neurological impairments), and at least one of the following: 1) a white blood cell count > 100 cells/mm3, protein > 50 mg/dL, and glucose< 2.5 mmol/L in CSF; 2) positive findings on Gram staining of CSF; and 3) positive cultures of blood (Kong et al., 2022).

### 2.4 Collection of samples

Data regarding surgery descriptions, clinical manifestations, demographic characteristics, and preexisting medical conditions were collected. In patients with suspected intracranial infection, CSF cell count; detection of HBP, PCT, CRP, glucose, and protein; and CSF culture or (and) NGS (requiring the consent of the patient or family and testing at their own expense) were performed. Patients were divided into infected and noninfected groups based on blood culture or NGS results. The infected group was further divided into gram-positive and gram-negative bacterial groups. The primary endpoint was death, and the secondary endpoint was the absence of clinical symptoms of intracranial infection and a negative CSF culture.

CSF bacterial culture: Under strict aseptic procedures, a lumbar puncture was carried out to collect a sample of cerebrospinal fluid (CSF). The CSF was obtained from the patients and placed into sterile tubes. Then, it was centrifuged at 1000 × g for 10–15 minutes to sediment bacteria. The sedimented bacteria were cultured on blood agar and chocolate agar plates and incubated for 24–48 hours at 37°C in a candle jar. After incubation, bacteria were identified by their colony morphology. Phenotypic tests and limited biochemical tests, such as oxidase tests, indole tests, citrate tests, and TSI tests, were performed (Obaro et al., 2023).

For the NGS test, 3 to 5 mL of CSF samples were taken and transported through the cold chain to a third-party testing institution (Shanghai Reisai Biotechnology Co., Ltd.) for testing. The basic detection process includes sample collection, collection of cell-free DNA, library establishment, and biological information analysis. Pathogen detection by type is reported based on preestablished threshold criteria (Wilson et al., 2019). When the NGS results differ from the CSF bacterial culture results, CSF bacterial culture is performed again for a definitive diagnosis.

The level of HBP in cerebrospinal fluid (Heparin Binding Protein Assay, Hangzhou Zhonghan Shengtai Biotechnology Co., Ltd., China) was determined by a latex immunoturbidimetric assay on a Roche Cobas c702 automatic biochemical analyzer. The analytical sensitivity was 0.01 ng/mL. The detection range of HBP levels was 8–340 ng/ml. The level of PCT in cerebrospinal fluid was determined by the “Electrochemiluminescence Method” on the Cobas E601 electrochemiluminescence analyzer. The analytical sensitivity was 0.05 ng/mL. CRP was determined by latex-enhanced immunoturbidimetry. The instrument used was a Mindray crp-m100 specific protein immunoanalyzer (Shenzhen Mindray Biomedical Electronics Co., Ltd., China), and the kit used was a supporting kit.

### 2.5 Statistical methods

SPSS 22.0 statistical software was employed for analysis. The measurement data are presented as mean ± standard deviation ( $\bar{\text{x}}\pm \text{s}$ ). Analysis of variance (ANOVA) was utilized for multigroup comparisons, and the LSD-t test (least significant difference) was used for pairwise comparisons. Count data are expressed as n (%). The chi-square test was applied to compare the baseline characteristics. Receiver operating characteristic (ROC) curves were employed to analyze the value of each index in identifying pathogens. A p value less than 0.05 was considered statistically significant.

## 3 Results

### 3.1 Comparison of general data

There were no statistically significant differences in age, sex, body mass index(BMI), height, primary disease, and combined disease between any two of the three groups (p > 0.05) as shown in **Table 1**.

**Table 1:** Comparison of clinical data among gram-positive bacteria group, gram-negative bacteria group, and non-infected group [n(%), ( $\bar{\text{x}}$ ±s)].

Variables	G- bacterial group (n=142)	G+ bacterial group (n=128)	Non-infected group	Statistical test	*p* value
Sex(Male/Female)	64/78	60/68	54/66	0.117	0.943
Age (years)	46.34 ± 18.88	45.28 ± 19.22	44.89 ± 17.67	2.145	0.315
BMI (kg/m^2^)	22.88 ± 1.95	23.07 ± 2.04	22.41 ± 1.85	1.550	0.214
Height (cm)	164.83 ± 7.24	165.48 ± 8.12	165.95 ± 7.87	0.491	0.612
protopathy				1.247	0.975
Intracranial tumor	44 (30.99)	42 (32.81)	36 (30.00)		
craniocerebral trauma	53 (37.32)	51 (39.84)	48 ((40.00)		
Spontaneous cerebral hemorrhage	31 (21.83)	26 (20.31)	24 (20.00)		
arachnoid cyst	14 (9.86)	9 (7.03)	12 (10.00)		
Combined diabetes	18 (12.68)	12 (9.38)	6 (5.00)	3.893	0.147
Combined hyperlipidemia	8 (5.63)	14 (10.94)	5 (4.17)	4.273	0.111
Combined hypertension	2 (1.41)	6 (4.69)	3 (2.50)	2.543	0.280
Combined cardiopathy	8 (5.63)	4 (3.13)	5 (4.17)	0.945	0.624

G- bacteria group, Gram-negative bacteria group; G+ bacteria group, Gram-positive bacteria group; BMI, Body mass index.

### 3.2 Pathogen distribution

The distribution of pathogens associated with intracranial infection in 270 patients with intracranial infection is as follows. There are 140 strains of gram-negative bacteria (52.59%, including Pseudomonas aeruginosa, Acinetobacter baumannii, and Klebsiella pneumoniae), and 128 strains of gram-positive bacteria (47.41%, including coagulase-negative Staphylococcus, Staphylococcus aureus, and Enterococcus faecalis) (**Table 2**). No patients with multiple intracranial infections were identified in this study.

**Table 2:** Distribution and composition of bacterial strains cultured from the CSF of patients with bacterial intracranial infection.

Pathogenic bacteria	Number of strains (n)	Constituent ratio (%)
G- bacteria	142	52.59
*Pseudomonas aeruginosa*	67	24.81
*Acinetobacter baumannii*	37	13.70
*Klebsiella pneumoniae*	34	12.59
*E. coli*	2	0.74
*Enterobacter cloacae*	1	0.37
*Serratia marcescens*	1	0.37
G+ bacteria	128	47.41
*Coagulase negative staphylococcus*	60	22.22
*Staphylococcus aureus*	41	15.18
*Enterococcus faecium*	24	8.89
*Micrococcus*	2	0.74
*Streptococcus mitis*	1	0.37
Total	270	100

G- bacteria group, Gram-negative bacteria group; G+ bacteria group, Gram-positive bacteria group.

### 3.3 Comparison of serum inflammatory indicators

The levels of white blood cell(WBC), CRP, and PCT in the intracranial infection group and the non-infection group were compared. It was found that the levels of WBC count, CRP, and PCT in the intracranial infection group were higher than those in the non-infection group, with a statistical difference (P< 0.05) (**Table 3**).

**Table 3:** Comparison of serum inflammatory indexes between intracranial infection group and non-infection group.

	Serum PCT(ng/mL)	WBC(×10^9^/L)	CRP(mg/L)
Intracranial infection group	1.48 ± 0.11	10.16 ± 3.18	12.07 ± 2.98
Non-infected group	0.32 ± 0.04	4.51 ± 0.63	4.32 ± 1.46
*t* value	6.295	4.288	6.208
*p* value	<0.05	<0.05	<0.05

### 3.4 Comparison of HBP and other inflammatory indexes in CSF.

**Figure 2:**
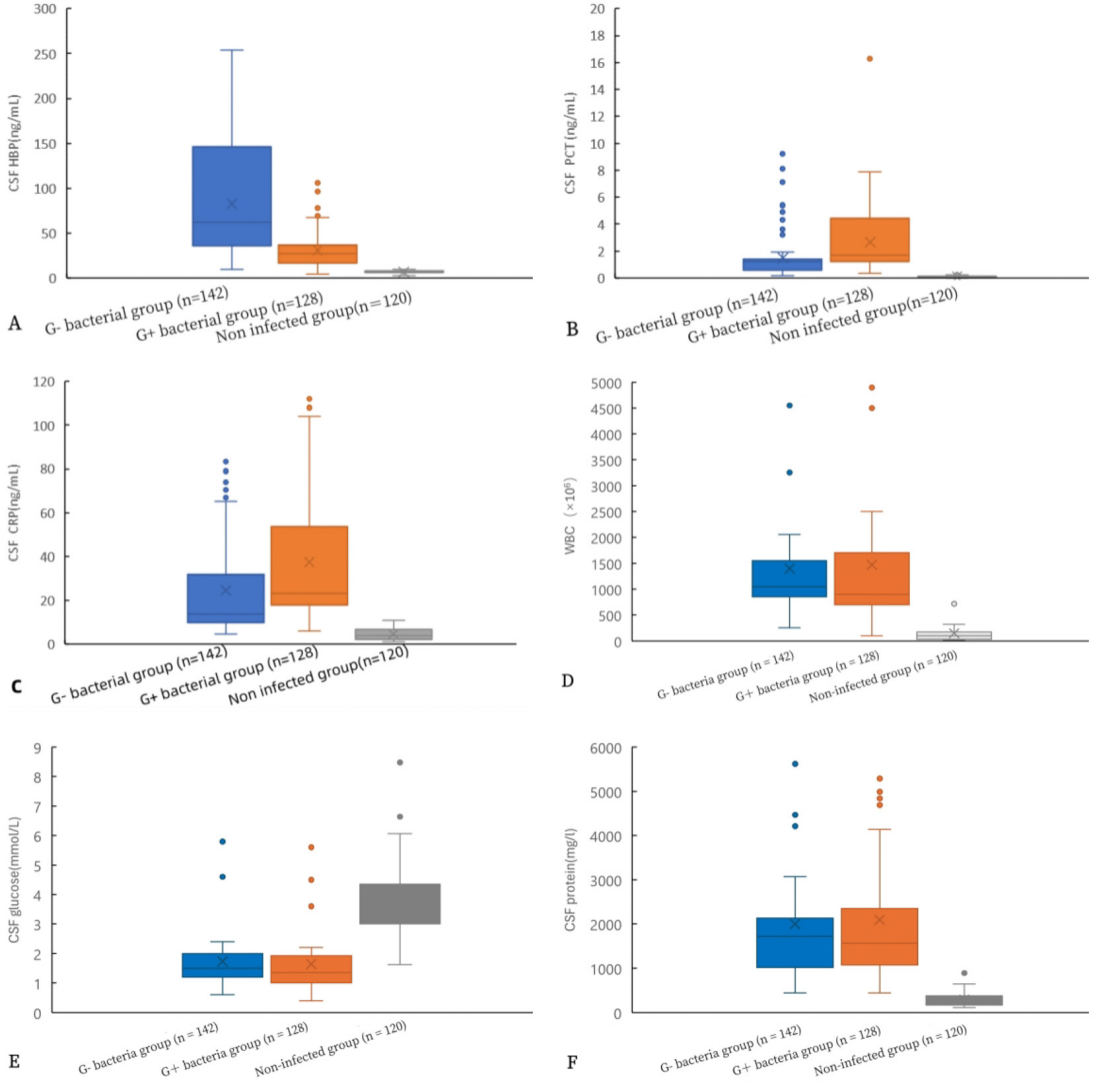
Comparison of HBP levels and other indicators in the CSF of gram-positive bacteria, gram-negative bacteria, and non-infected groups. HBP **(A)**, procalcitonin **(B)**, CRP **(C)**, WBC **(D)**, glucose **(E)**, and protein **(F)**.

**Figure 2** shows a comparison of various indicators in the CSF. Significantly different levels of HBP, PCT, CRP, cell count, glucose, and CSF protein were observed between the gram-positive bacteria group and the uninfected group, as well as between the gram-negative bacteria group and the uninfected group (p< 0.05). Additionally, there were statistical differences in HBP, PCT, and CRP levels between the gram-negative bacteria group and the gram-positive bacteria group (all p< 0.05). However, no statistical differences were found in cell count, glucose, and protein between these two bacterial groups (all p > 0.05).

### 3.5 Correlation between CSF traditional markers and HBP level

**Table 4** shows a significant correlation between CSF HBP and other CSF levels (WBCs, PCT, protein, and CRP).

**Table 4:** Correlation between CSF traditional markers and HBP level.

Traditional CSF markers	CSF WBCs count/mm^3^	CSF Protein (mg/dl)	CSF PCT	CSF CRP
CSF HBP	r=0.351, p<0.001	r=0.575, p<0.001	r=−0.586, p< 0.001	r=0.664, p< 0.001

At the same time, the correlation between PCT in serum and PCT in CSF was analyzed. There was a strong positive correlation between PCT in serum and PCT in CSF (r = 0.976, P< 0.001).

### 3.6 NGS detection and microbiological culture results

A total of 60 patients with intracranial infection underwent simultaneous detection by metagenomic next-generation sequencing (mNGS) and cerebrospinal fluid (CSF) culture. Among them, 50 patients were positive for mNGS, with a detection rate of 83.00%. Pathogenic microorganisms were detected in 36 patients by bacterial culture, with a detection rate of 60.00%. The detection rate of pathogenic microorganisms in patients by mNGS was higher than that by bacterial culture (P< 0.05).

### 3.7 ROC curve analysis

**Figure 3:**
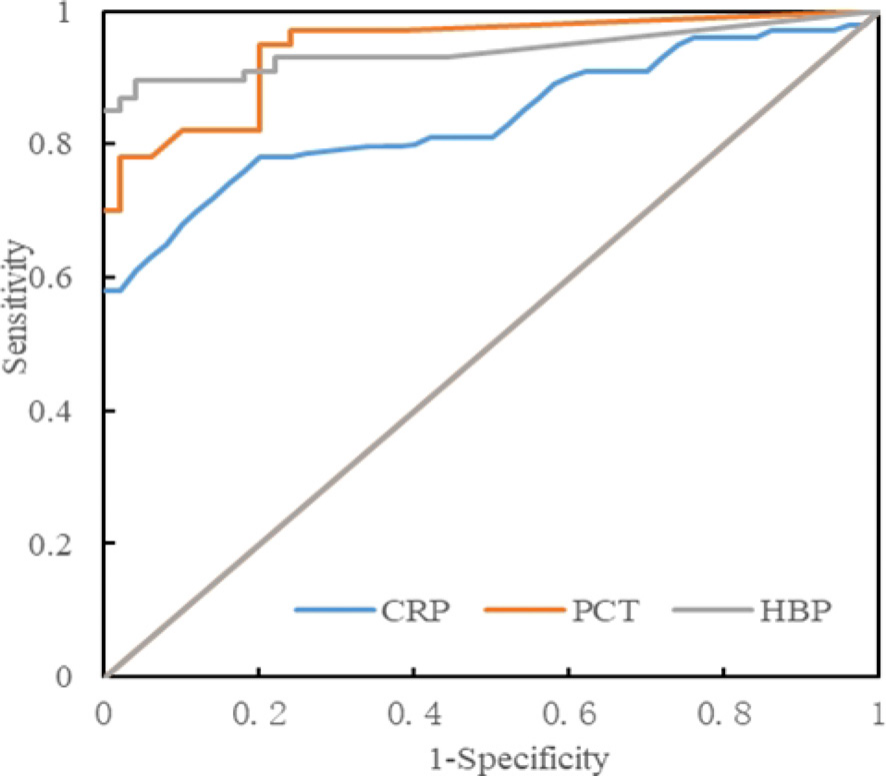
ROC curve analysis of the intracranial infection group and the non-infected group.

The levels of HBP, PCT, and CRP in the infected group and the non-infected group were compared, and ROC curves were drawn. The area under the curve (AUC) of HBP, PCT, and CRP were 0.935, 0.931, and 0.863 respectively, all of which were greater than 0.7. When the specificity of HBP, PCT, and CRP was 100%, their cutoff values were 15.80 ng/L, 0.29 ng/L, and 10.12 ng/L respectively (**Table 5**, **Figure 3**). When the cutoff value of PCT was 0.27, its sensitivity and specificity were 78.3% and 98.3%. And when the cutoff value of CRP was 9.45, its sensitivity and specificity were 78.3% and 91.7%.

**Table 5:** Diagnostic efficacy of each index between the infected group and the non-infected group.

Index	AUC	Cutoff value	95%CI	sensitivity(%)	specificity(%)	*p* value
HBP (ng/mL)	0.935	15.80	0.902-0.970	85.0	100.0	<0.05
PCT (ng/mL)	0.931	0.29	0.896-0.968	70.2	100.0	<0.05
CRP (ng/mL)	0.863	10.12	0.831-0.899	58.0	100.0	<0.05

**Figure 4:**
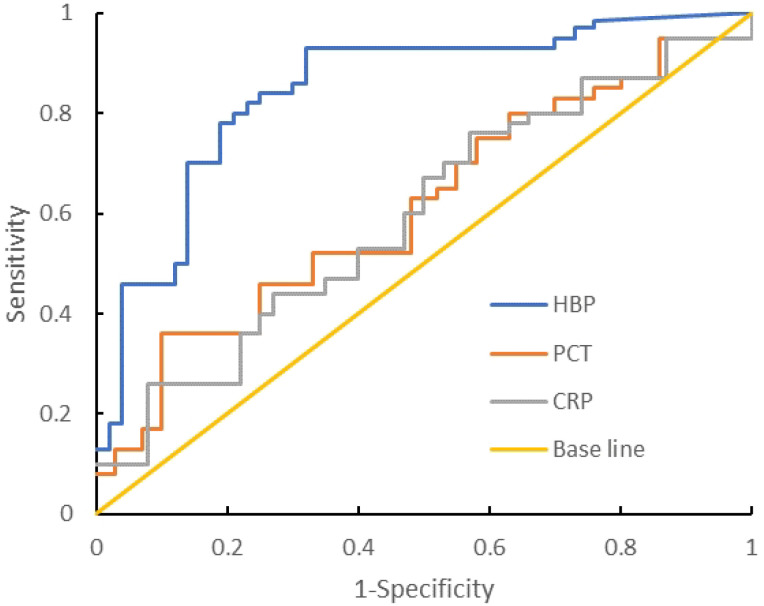
ROC curve analysis of the Gram-negative bacteria group and Gram-positive bacteria group.

Compared with the Gram-negative bacteria and Gram-positive bacteria groups, the AUC of HBP, PCT, and CRP were 0.816, 0.602, and 0.691 respectively. The AUC of HBP was the largest. When the cutoff value of HBP is 34.80 ng/mL, its sensitivity and specificity are 92.3% and 69.1%. When the cutoff value is 72.34 ng/mL, its sensitivity and specificity are 57.8% and 96.1%. When the cutoff value of PCT is 1.67 ng/mL, its sensitivity and specificity are 52.3% and 66.9%. When the cutoff value of CRP is 23.12 ng/mL, its sensitivity and specificity are 53.1% and 59.9% (**Table 6**, **Figure 4**).

**Table 6:** Diagnostic efficacy of each indicator between Gram-positive bacteria group and Gram-negative bacteria group.

Index	AUC	Cutoff value	95% *CI*	sensitivity(%)	specificity(%)	*p* value
HBP (ng/mL)	0.816	72.34	0.734-0.899	57.8	96.1	<0.05
PCT (ng/mL)	0.602	1.67	0.469–0.730	52.3	66.9	<0.05
CRP (ng/mL)	0.591	23.12	0.457–0.725	53.1	59.9	<0.05

We also studied the diagnostic efficacy of serum C-reactive protein (CRP) and procalcitonin (PCT) levels in differentiating between gram-negative bacteria and non-gram-negative bacteria. The area under the AUC of serum CRP was 5.132, with a specificity of 45.2% and a sensitivity of 87%. The AUC of serum PCT was 5.246, with a specificity of 48.6% and a sensitivity of 85.5%. The diagnostic efficacy of serum PCT and CRP was lower than that of CSF.

## 4 Discussion

HBP, PCT, and CRP are commonly used indicators for predicting infectious diseases in clinical practice and have high diagnostic value in various bacterial infections (Kong et al., 2023; Norman-Bruce et al., 2024). However, there are few reports on the detection of HBP, PCT, and CRP in CSF for intracranial infection. In this study, the levels of CSF HBP, PCT, and CRP in the intracranial infection group were greater than those in the non-infection group. The AUCs of CSF HBP, PCT, and CRP for the diagnosis of intracranial infection were 0.935, 0.931, and 0.863 respectively. These findings suggest that the levels of HBP, PCT, and CRP in the CSF can accurately predict intracranial infection. Linder et al (Linder et al., 2011). reported that when the cutoff value was 20 ng/mL, the AUC of CSF HBP for diagnosing bacterial intracranial infection was as high as 0.994, and the sensitivity and specificity for diagnosing bacterial intracranial infection were 100% and 99.2% respectively. The results of this study revealed that when the optimal cutoff value was 15.80 ng/mL, the AUC of CSF HBP for diagnosing bacterial intracranial infection was 0.935, and its sensitivity and specificity were 85.00% and 100% respectively. Although there are some differences in the results, which may be related to the sample size, selection of cutoff values, and the use of antibiotics, all indicate that HBP can be used as an early diagnostic indicator of intracranial bacterial infection. In this study, the sensitivity and specificity of PCT in diagnosing intracranial bacterial infection were 78.3% and 98.3% respectively, which were somewhat different from the 69% and 100% reported by Schwarz et al (Schwarz et al., 2000). and the 86% and 80% calculated by Wei et al (Wei et al., 2016), which may be related to the sample size. However, PCT is an important marker of intracranial bacterial infection. In this study, the sensitivity and specificity of CRP were 69.2% and 91.7% respectively, which were somewhat different from the 93.4% and 86.4% reported by Lu et al (Lu et al., 2021). This may be related to the selection of cutoff values and sample sizes. When the specificity of HBP, PCT, and CRP was 100%, their cutoff values were 15.80 ng/L, 0.29 ng/L, and 10.12 ng/L respectively, and the sensitivities were 85.0%, 70.2%, and 58.0%. The results indicated that when the HBP level was greater than 15.80 ng/mL, the patient was considered to have intracranial bacterial infection. When the PCT level was greater than 0.29 ng/mL or CRP was greater than 10.12 mg/L, the patient was considered to have intracranial bacterial infection.

This study also analyzed the distribution of CSF bacterial culture strains associated with intracranial infection, which is roughly the same as that reported by Zhang et al (Zhang et al., 2021). Gram-negative bacteria accounted for 52.59% of intracranial bacterial infections, and Gram-positive bacteria accounted for 47.41%. Among the Gram-negative bacteria, Pseudomonas aeruginosa accounted for 24.81%, Acinetobacter baumannii accounted for 13.70%, and Klebsiella accounted for 12.59%. The Gram-positive bacteria included coagulase-negative Staphylococcus (22.22%), Staphylococcus aureus (15.18%), and Enterococcus faecium (8.89%).

We compared the levels of HBP, PCT, and CRP in the Gram-negative bacteria group and the Gram-positive bacteria group. The comparison of HBP in the Gram-negative bacteria and Gram-positive bacteria groups showed that the AUC was 0.816, while the AUCs of PCT and CRP were 0.602 and 0.591 respectively, indicating that HBP is superior to PCT and CRP in the comparison of Gram-negative and Gram-positive bacteria groups. When the cutoff value of HBP is 72.34 nm/mL, its specificity is 96.1% and its sensitivity is 57.8%. When HBP is >72.34 nm/mL, it has diagnostic value for Gram-negative bacteria intracranial infection. However, its sensitivity is low and there will be more missed diagnoses. It is necessary to combine other predictors to improve sensitivity and reduce missed diagnoses. The mechanism of increased HBP level in intracranial infection with Gram-negative bacteria may include: (1) the antagonistic effect of HBP on Gram-negative bacteria. After Gabay et al (Gabay et al., 1989). isolated azurol, they reported that it is produced by neutrophils against Gram-negative bacteria. HBPs can be used as chemical attractants (activators of monocytes/macrophages). Through the neutrophil β integrin junction, it triggers vascular leakage and edema. (2) As a chemokine, HBP can activate monocytes/macrophages and differentially regulate endotoxin-induced TNF-α, increasing the levels of proinflammatory cytokines such as IL-1 and IL-6. In this study, Gram-positive bacteria had higher PCT and CRP levels than Gram-negative bacteria. When PCT and CRP levels were less than 1.67 nm/mL and 23.12 nm/mL respectively, the sensitivities (52.3%, 53.1%) and specificities (66.9%, 59.9%) were both low, and the diagnostic value for Gram-negative bacteria was not great.

To the best of our knowledge, this study is the first to utilize HBP in cerebrospinal fluid to distinguish between intracranial Gram-negative bacterial infection and Gram-positive bacterial infection. The disadvantage is that our study sample comprised patients with intracranial infection due to various reasons, and further analysis of whether antibiotics were used before lumbar puncture was not carried out. This study is the first to use HBP in cerebrospinal fluid to distinguish between intracranial Gram-negative bacterial infection and Gram-positive bacterial infection. Serum HBP was not studied in this study, but it did not have a conclusive influence on the results of the study.

## 5 Conclusions

HBP, PCT, and CRP can be employed as differential indicators for intracranial bacterial infection (HBP > 15.80 ng/mL, PCT > 0.29 ng/mL, CRP > 10.12 ng/mL). Among them, HBP > 72.34 ng/mL can serve as an independent index for the diagnosis of Gram-negative bacteria infection. Nevertheless, its sensitivity is low, and there will be a greater likelihood of missed diagnoses. It is necessary to combine it with other predictors to enhance sensitivity and reduce missed diagnoses. HBP is a promising biomarker for intracranial infection and can be used for the routine detection of patients with intracranial infection, providing a reference for clinicians, improving the cure rate of intracranial infection, and enhancing the prognosis.

## Data Availability

The original contributions presented in the study are included in the article/supplementary material. Further inquiries can be directed to the corresponding author/s.
